# Ccar1 prevents β-catenin nuclear translocation to sustain ground-state pluripotency in mouse ESCs under R2i

**DOI:** 10.1016/j.bbrep.2025.102246

**Published:** 2025-09-07

**Authors:** Sara Taleahmad, Hossein Abbasinia, Azam Samadian, Sepideh Molamohammadi, Hossein Baharvand

**Affiliations:** a Department of Stem Cells and Developmental Biology, Cell Science Research Center, Royan Institute for Stem Cell Biology and Technology, ACECR, Tehran, Iran; b Department of Developmental Biology, University of Science and Culture, ACECR, Tehran, Iran

**Keywords:** Mouse embryonic stem cells, TGFβ inhibition, R2i, MEK inhibition

## Abstract

Dual inhibition of extracellular signal-regulated kinase (MEK) and transforming growth factor beta (TGFβ), known as R2i, sustains ground-state pluripotency in mouse embryonic stem cells (mESCs). To understand the molecular mechanisms of R2i, we analyzed the proteomic profile of mESCs cultured under R2i conditions in our previous study. Our data showed overexpression of the cell cycle and apoptosis regulator 1 (Ccar1) under R2i conditions. In this study, we investigated the role of Ccar1 in the pluripotency of mESCs through the loss-of-function approach. We hypothesize that Ccar1 contributes to the maintenance of pluripotency by interacting with β-catenin and preventing its translocation to the nucleus. Therefore, we used siRNA against Ccar1 and then analyzed the localization of β-catenin and the expression of its target genes by immunofluorescence assay and qRT-PCR. Immunofluorescence analysis demonstrated that siRNA-mediated downregulation of Ccar1 promoted the nuclear translocation of β-catenin. qRT-PCR analysis showed a significant reduction of pluripotency marker genes as well as some cell cycle markers such as *Ccar1*, *c-myc*, and *Tbx3* in siRNA-treated cells. In addition, the expression level of the Wnt target genes (*Cdx1*, *Wnt3a*, *Tbx1*, *Fgf4*, *Apc*, *Cdh1*, *Wnt3a*) was significantly increased when Ccar1 was knocked down. We observed that upregulation of Ccar1 under R2i culture conditions could prevent nuclear translocation of β-catenin and maintain pluripotency and self-renewal of mESCs. These findings suggest that Ccar1 prevents nuclear β-catenin translocation to maintain pluripotency and self-renewal of mESCs under R2i conditions, although further direct interaction assays are required to confirm this mechanism.

## Highlights

• Ccar1 upregulation maintains pluripotency in mESCs under R2i conditions.
• Loss of Ccar1 disrupts β-catenin localization and reduces pluripotency markers.
• Knockdown of Ccar1 leads to increased expression of Wnt target genes.
• Immunofluorescence confirms Ccar1's role in preventing β-catenin nuclear translocation.
• Study highlights Ccar1 as a key regulator of mESC pluripotency and self-renewal.

## 1 Introduction

Mouse embryonic stem cells originate from the inner cell mass of blastocysts and can retain their ability to differentiate into multiple cell types when cultured with certain factors, such as leukemia inhibitory factor (LIF), bone morphogenetic protein 4 (BMP4), and chemicals that inhibit differentiation signals. Smith and colleagues reported that dual inhibition of fibroblast growth factor 4 (FGF4) and glycogen synthase kinase 3 (GSK3) by PD0325901 and CHIR99021, known as 2i, plays a key role in determining the ground state of pluripotency [1]. Another study showed that inhibition of mitogen-activated protein kinase and transforming growth factor β with PD0325901 and SB431542, known as R2i, can effectively generate mouse embryonic stem cells [2,3]. In our previous study, we reported that R2i treatment causes an increase in cell division cycle and apoptosis regulatory protein 1 (Ccar1/Carp1) compared to serum and 2i conditions [4]. CCAR1 is a transcriptional coactivator that interacts with β-catenin and enhances the ability of β-catenin to activate target genes of the Wnt signaling pathway [5]. The Wnt/beta-catenin pathway is known for its involvement in embryonic development, stem cell maintenance, and tissue regeneration.

If there is a documented interaction between CCAR1 and beta-catenin, it could suggest a potential cross-talk between cell cycle regulation, apoptosis, and the Wnt signaling pathway. In this regard, it has been shown that circZKSCAN1 suppresses hepatocellular carcinoma stem cells by regulating the activity of RBP-sensitive X mental retardation protein (FMRP), whose downstream target gene is CCAR1, and showed that CCAR1 acts as a co-activator of the Wnt/β-catenin signaling pathway and is critical for generating an optimal TGF-β signaling response [6].

In addition, Wang, L et al. established 5-fluorouracil-resistant cell lines and investigated potential targets associated with 5-fluorouracil resistance in advanced colorectal cancer. They found that double cortin-like kinase 1 (DCLK1) interacts with cell cycle and apoptosis regulator 1 (CCAR1) through its C-terminal domain and phosphorylates CCAR1 at Ser343, which is essential for CCAR1 stabilization. In addition, they found that DCLK1 positively regulates β-catenin signaling through CCAR1, which is responsible for the maintenance of cancer stem cells. Importantly, they showed that DCLK1 inhibitor can block cancer stem cells mediated by the CCAR1/β-catenin signaling pathway and thus suppress 5-fluorouracil-resistant CRC cells *in vitro* and *in vivo* [7]. It has been reported that stabilizing β-catenin in the cytoplasm promotes self-renewal in mouse EpiSC and human ESC, while nuclear translocation of β-Catenin and its binding to TCFs stimulate differentiation [8]. More recent studies further demonstrated that Wnt/β-catenin activity has distinct nuclear and cytoplasmic roles in regulating the balance between self-renewal and differentiation.

These data prompted us to investigate the interaction between Ccar1 and β-catenin and the role of this in the maintenance of pluripotent stem cells under R2i conditions. Understanding the specific target genes and signaling pathways influenced by this interaction provides valuable insights into the molecular mechanisms governing self-renewal. Here, we show that Ccar1 is involved in the pluripotency maintenance of R2i-grown cells, and that the suppression of its expression results in a decreased pluripotency marker gene.

## 2 Materials and methods

### 2.1 mESCs culturing

The mESC lines in independent biological replicates (Royan B20, Royan Institute) were cultured in 2i/Leukemia Inhibitory Factor (LIF), R2i/LIF (serum-free N2B27 medium), and serum/LIF medium on 0.1 % gelatin-coated plates (Sigma-Aldrich Cat. No. 9000-70-8). The 2i medium contained the MEK and GSK3 inhibitors PD0325901 (1 μM; Stemgent) and CHIR99021 (3 μM; Stemgent)1. The R2i culture consisted of 1 μM PD0329501 and 10 μM SB431542, which inhibit the TGFβ signaling pathway [3]. N2B27/LIF medium consisted of DMEM/F12 (Invitrogen) and Neurobasal (Invitrogen) in a 1:1 ratio, 1 % B27 supplement (Invitrogen), 1 % N2 supplement (Invitrogen), 2 mM l-glutamine (Invitrogen), 0.1 mM β-mercaptoethanol (Sigma-Aldrich Cat. 60-24-2), 1 % non-essential amino acids (Invitrogen), 100 mg/ml streptomycin (Invitrogen), 100 U/ml penicillin (Gibco Cat. 8025-06-7) and 5 mg/ml bovine serum albumin (BSA; Sigma-Aldrich Cat. 9048-46-8). The serum medium contained Knockout Dulbecco’s modified Eagle’s medium (KoDMEM; Invitrogen), 100 mg/ml streptomycin (Gibco Cat. No. 8025-06-7), 2 mM l-glutamine (Gibco), 1 % non-essential amino acids (Sigma Cat. No. M7145), 15 % fetal bovine serum (FBS; HyClone Sigma Cat. No. F7524), 100 U/ml penicillin and 0.1 mM β-mercaptoethanol (Sigma Cat. No. 60-24-2).

### 2.2 Immunofluorescence (IF) staining and alkaline phosphatase (ALP) detection

We performed immunofluorescence after fixation of the cultured cells in 4 % paraformaldehyde (Sigma Cat No. 30525-89-4) for 20 min, permeabilization with 0.2 % Triton X-100 (Merk Cat No. 9036-19-5) for 30 min, and blocking in 10 % secondary antibodies host serum in PBS for 1 h. The cells were incubated with the primary antibody overnight at 4 °C. After washing, the cells were subsequently incubated with the secondary antibody for 1 h at 4 °C. The cells were stained with 4’,6-diamidino-2-phenylindole (0.1 mg/ml; Sigma, D8417 Cat No. 28718-90-3) for 10 min in the dark, and after washing, they were analyzed using an Olympus fluorescent microscope (Olympus, Japan). Alkaline phosphatase (ALP) activity was detected based on the manufacturer’s instructions (Sigma-Aldrich, 86R).

### 2.3 qRT-PCR analysis

Total RNA was extracted from three biological replicates of 2i, R2i, and serum-cultured cells using the RNeasy Mini Kit (Qiagen) according to the manufacturer’s instructions. The purity and concentration of total RNA were quantified using a Biowave II spectrophotometer (WPA, Biochrom). The quality and integrity of total RNA were confirmed by electrophoresis on a 1 % agarose gel stained with GelRed. cDNA was prepared using the RevertAid cDNA synthesis kit and random hexamer primers (Thermo Fisher Scientific) according to the manufacturer’s instructions. Transcript levels were determined using the SYBR Green Master Mix (ABI) on the Applied Biosystem Real Time PCR System (ABI, Step One plus, USA). The results were normalized to the GAPDH housekeeping gene and the relative quantification of gene expression was calculated using the ΔΔ^ct^ method.

### 2.4 Western blot analysis

Cells were lysed with lysis buffer according to the manufacturer’s instructions (TRIzol Reagent, Ambion). Total protein (50 μg) was separated on a 10 % SDS-polyacrylamide gel and transferred onto a PVDF membrane (Bio-Rad). The blots were blocked in TBS solution containing 20 mM Tris-HCl (pH 7.6, 150 mM NaCl),0.1 % Tween-20, and 5 % BSA. The membranes were incubated overnight at 4 °C with primary antibody solution (Ccar1; Santa Cruz, sc-515629 and β-Catenin; Santa Cruz, sc-59737). After washing, Ccar1 and β-Catenin proteins were detected with horseradish peroxidase (HRP)-conjugated secondary antibody. Signals were recorded with an ECL substrate (GE) using Hyper-film (GE).

### 2.5 siRNA transfection

We employed ON-TARGET plus SMART pool siRNAs designed to target Mouse sequences. R2i cells were seeded in a six-well tissue culture plate at 80 % confluency. The transfection of siRNA was carried out using Lipofectamine TM 2000 (Invitrogen) as per the manufacturer's instructions. A total of 50 nM siRNA from a pool of Ccar1 siRNAs and scramble siRNA (si-Ctrl), along with 5 μL of Lipofectamine-3000 reagent (Invitrogen) and 100 μL of RPMI, were preincubated for 20 min. This mixture was then combined with 900 μL of RPMI to create the transfection mixture. After 48 h, cells treated with siCcar1 and si-Ctrl from three biological replicates were harvested for cell cycle and molecular analyses. The efficacy of siRNA transfection was assessed using FITC-conjugated siRNA (Invitrogen).

### 2.6 Statistical evaluation

All data were expressed as mean ± S.D (standard deviation) and statistical analysis was performed using one-way analysis of variance (ANOVA) test, followed by a Tukey *post-hoc* test. For the determination of significant differences among groups, P < 0.05 was considered statistically significant.

## 3 Results

### 3.1 Up-regulation of cell division cycle and apoptosis regulator protein 1 under R2i conditions

**Fig. 1:**
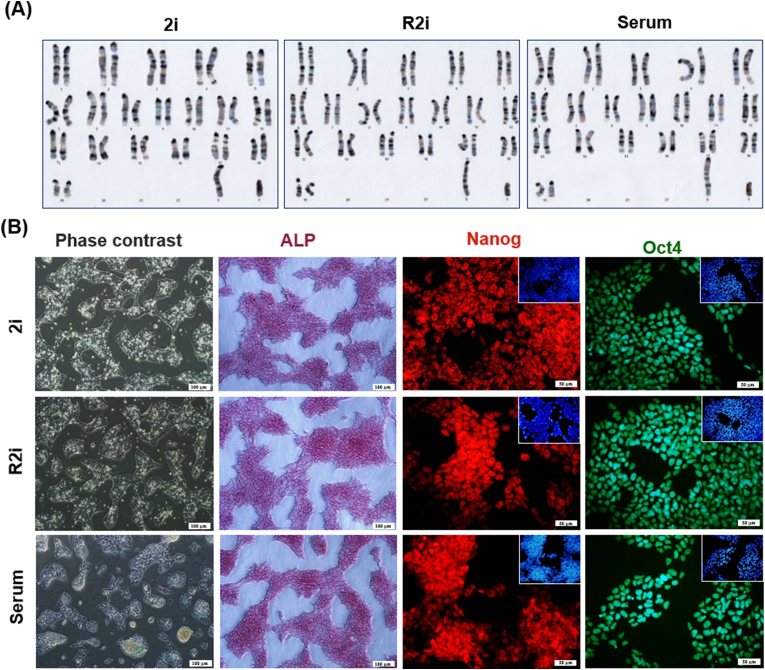
**Characteristics of the mouse ES cells cultivated in 2i, R2i, and serum. (A)** Karyotype **(B)** Phase contrast, alkaline phosphatase (ALP) staining (Scale bar = 100 μm) and immunofluorescence labeling for Oct4 and Nanog, counterstained for DAPI, are shown (Scale bar = 50 μm).

**Fig. 2:**
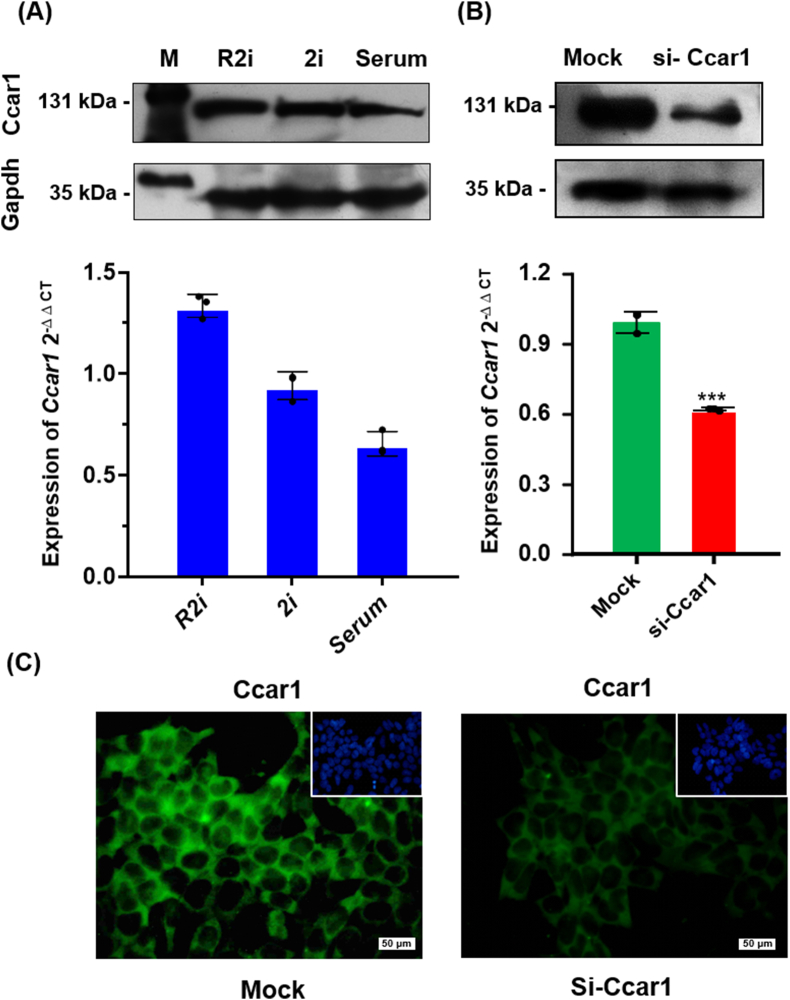
**Expression level of Ccar1 under 2i-, R2i- and serum-grown cells. (A)** qRT-PCR and Western blot analyses of the *Ccar1* under 2i-, R2i- and serum-grown cells. **(B)** qRT-PCR and Western blot analyses of the *Ccar1* under mock and siRNA-treated cells. (qRT-PCR; n = 3, ∗∗∗p < 0.001). Each mRNA expression level in the cells was normalized to the *Gapdh* housekeeping gene. **(C)** Immunostaining assay of Ccar1 under mock and siRNA-treated cells.

According to our previous analysis of proteomics data [4], we demonstrated that the upregulation of cell division cycle and apoptosis regulator protein 1 (Ccar1) was significantly higher under R2i conditions compared to serum and 2i conditions, 5.6- and 2.6-fold, respectively. Morphology and pluripotency characterization of cells grown under 2i, R2i and serum conditions showed normal karyotype (Fig. 1A) and phase contrast, alkaline phosphatase activity (ALP), as well as expression of key mouse ESC markers, including Oct-4, and Nanog (Fig. 1B). To confirm these results, we examined the expression of Ccar1 under the three culture conditions and found that Ccar1 was upregulated in R2i compared to 2i and serum at both mRNA and protein levels (Fig. 2A).

### 3.2 Ccar1 can interact with β-catenin under R2i conditions

Previous studies have shown that CCAR1 can interact with β-catenin in human clonal carcinoma cells and enhance its ability to activate transfected reporter genes [5]. However, there is currently no evidence of interaction between mouse Ccar1 and β-catenin proteins. To investigate this, we performed an *in-silico* analysis in which human and mouse Ccar1 and β-catenin sequences were aligned and compared. Their analysis showed that the human and mouse β-catenin sequences matched more than 99 % and the human and mouse Ccar1 sequences matched 94.8 %. To investigate whether Ccar1 associates with β-catenin, by the loss-of-function approach, we used specific siRNA against *Ccar1* and examined the function of β-catenin under R2i conditions. The efficiency of siRNA treatment was confirmed at both the mRNA and protein levels by qRT-PCR and Western blot analyses, respectively (Fig. 2B). Knockdown of *Ccar1* was also confirmed by immunocytochemical assay (Fig. 2C).

**Fig. 3:**
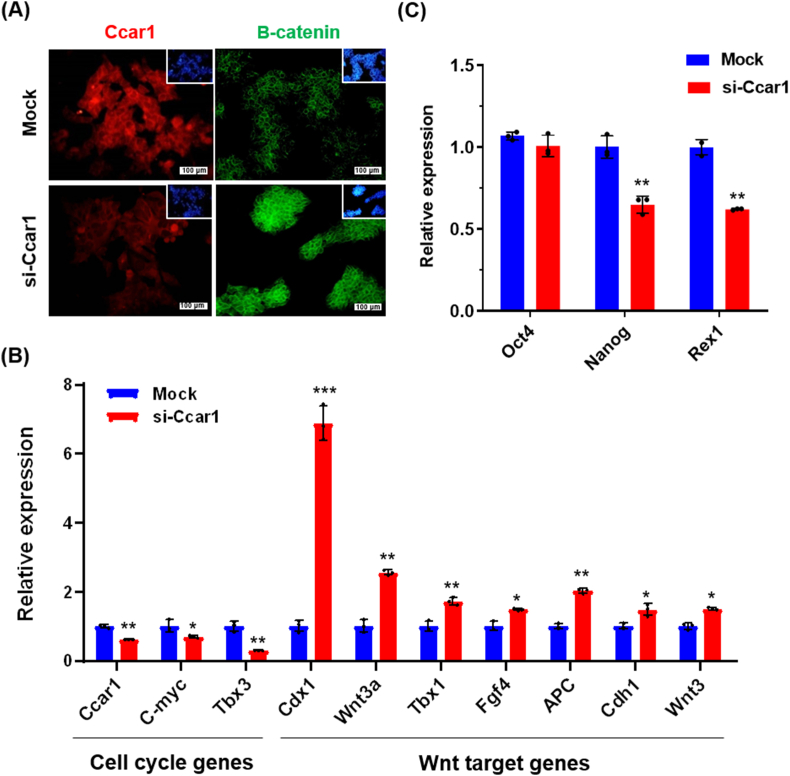
**The effect of si-*Ccar1* treatment on the expression of related genes. (A)** Immunostaining assay of Ccar1 and β-catenin under mock and siRNA-treated cells. **(B)** The expression levels of cell cycle genes (*Ccar1*, *C-myc,* and *Tbx3*) and Wnt signaling target genes (*Wnt3a*, Tbx1, *Fgf4*, *APC*, *Cdh1,* and *Wnt3*) were quantified by real-time PCR in si-Ccar1-treated cells. **(C)** The expression level of pluripotency marker genes *Oct4*, *Nanog* and *Rex1* in si-Ccar1 treated *cells*. (qRT- PCR; n = 3, ∗p < 0.05, ∗∗p < 0.01, ∗∗∗p < 0.001). Each mRNA expression level in the cells was normalized to the *Gapdh* housekeeping gene.

Next, we examined β-catenin localization following siRNA treatment. We observed a nuclear translocation of β-catenin under si-*Ccar1* treatment using immunostaining (Fig. 3A).

Consistent with this nuclear shift, quantitative PCR revealed a significant increase in the expression of canonical Wnt/β-catenin target genes (*Cdx1*, *Wnt3a*) and a decrease in naïve pluripotency genes (*Nanog*, *Rex1*) following Ccar1 knockdown. These transcriptional changes are in agreement with β-catenin’s known role in promoting differentiation when localized to the nucleus, supporting an indirect functional link between Ccar1 and β-catenin in sustaining pluripotency under R2i.

### 3.3 Suppression of *Ccar1* reduces the expression of cell cycle target genes

As *Ccar1* is a cell cycle regulator, we investigate the expression of two important genes (*Tbx3* and *C-myc*) in this signaling. Our data indicated that the expression of *Tbx3* and *C-myc* was reduced under si-RNA-treated cells (Fig. 3B). *Tbx3* and *C-myc* are both implicated in cell cycle regulation and cellular proliferation. The reduction in their expression following *Ccar1* knockdown could indicate that *Ccar1* positively regulates pathways associated with cell cycle progression, potentially through direct or indirect interactions with these genes.

### 3.4 Nuclear translocation of β-catenin correlated with Wnt target gene expression

We observed that inhibition of the interaction between β-catenin and Ccar1 by si-*Ccar1* treatment leads to nuclear translocation of β-catenin, where it forms complexes with DNA-binding proteins such as TCFs to activate transcription. To investigate whether the nuclear translocation of β-catenin was affected by si-*Ccar1* treatment, we analyzed its subsequent binding to TCFs by examining the expression of Wnt target genes. We found that knockdown of *Ccar1* increased the expression of *Cdx1*, *Wnt3a*, *Tbx1*, *Fgf4*, *APC*, *Cdh1*, and *Wnt3* (Fig. 3B). This pattern is consistent with β-catenin/TCF transcriptional activation following nuclear translocation in the absence of Ccar1.

### 3.5 Cytoplasmic β-catenin mediates R2i-grown cells ‘self-renewal

Besides examining the effects of reduced *Ccar1* on the Wnt signaling target genes, we also investigated Ccar1 function on the influence of cytoplasmic stabilization of β-catenin on the self-renewal of R2i conditions. We showed that the expression of *Nanog* and *Rex1* was decreased under si-*Ccar1* treatment compared to mock, but there was no significant change in the expression of *Oct4* (Fig. 3C). Together with Wnt target induction, these data suggest a partial exit from ground-state pluripotency/self-renewal under R2i when Ccar1 is reduced, rather than a complete differentiation program. The decreased expression of *Nanog* and *Rex1* under si-*Ccar1* treatment suggests that the knockdown of *Ccar1* and subsequent nuclear translocation of β-catenin may have a negative impact on the self-renewal capacity of R2i-grown cells. This finding suggests that Ccar1 may play a role in maintaining the self-renewal and pluripotency of mouse embryonic stem cells under R2i conditions by regulating the localization and activity of β-catenin.

## 4 Discussion

Maintenance of self-renewal in mouse embryonic stem cells (mESCs) is a highly intricate process, regulated by various factors. Among the key players in this intricate network, β-catenin, a protein known for its involvement in cell adhesion and Wnt signaling, has emerged as a pivotal regulator to create the balance between self-renewal and differentiation in mESCs. This paper explores the intriguing interaction between the cell cycle and apoptosis regulator 1 (Ccar1) and β-catenin, elucidating its role in the cytoplasmic stabilization of β-catenin and the prevention of its nuclear translocation.

Ccar1 was initially characterized as a protein that was implicated in cell growth inhibition and apoptosis signaling [9]. Our previous research indicated an increase in Ccar1 expression in cells cultured under R2i conditions [4,10]. We hypothesize that this heightened expression is linked to the maintenance of pluripotency in R2i-cultured cells. Additionally, it has been reported that Ccar1 can interact with β-catenin and stabilize it in the cytoplasm of hESCs. In cellular processes, β-catenin is a key mediator of the canonical Wnt signaling pathway, where it translocates into the nucleus to activate the transcription of target genes associated with cell proliferation and differentiation. It seems that cytoplasmic stabilization of β-catenin has a significant role in the self-renewal and pluripotency maintenance of ESCs. Therefore, by the loss-of-function approach, we used specific siRNA against Ccar1. Our data indicated downregulation of *Ccar1* in siRNA-treated cells. Also, the expression of *c-myc* and *Tbx3* was reduced in siRNA-treated cells. This data indicated that Ccar1 is involved in cell growth, and it has been reported that suppression of CCAR1 induced apoptosis in AGS and MKN28 cells [11]. This view accords with prior reports that cytoplasmic β-catenin supports self-renewal, whereas nuclear β-catenin/TCF activity can drive lineage programs in pluripotent cells [8].

Consistently, Ccar1 knockdown facilitated nuclear entry of β-catenin, leading to TCF/LEF-mediated activation of canonical Wnt target genes, including *Cdx1, Wnt3a, Tbx1, Fgf4, APC, Cdh1, and Wnt3.* Our results are consistent with those reported by Michael R. Stallcup et al. They investigate the role of Ccar1, which supports β-catenin in its gene-activating task. The authors provide an in-depth analysis of the molecular mechanisms. They saw that CCAR1 only goes to the genes targeted by Wnt when β-catenin is present. When they removed CCAR1 from the cells, they found that the genes targeted by Wnt were not properly activated, and the cancer cells did not grow as they should [5]. While Stallcup et al. identified CCAR1 as a β-catenin coactivator in carcinoma cells, our results suggest a distinct role in mESCs, where Ccar1 primarily prevents nuclear translocation of β-catenin. This discrepancy may reflect context-dependent functions of Ccar1, varying between cancer cells and pluripotent stem. “Furthermore, a recent study revealed that CCAR-1 collaborates with the U2AF large subunit UAF-1 to regulate alternative splicing, highlighting an unexpected role in post-transcriptional gene regulation beyond its known functions in signaling and transcriptional coactivation [12].

A study investigated the role of CCAR1 in several human cancers, including gastric cancer. The findings demonstrate that CCAR1 interacts with β-catenin and plays a crucial role in regulating pluripotency genes. The study further revealed that excessive nuclear activity of CCAR1 in gastric cells is associated with gastric cancer. They also found that reducing CCAR1 in these cells slows their growth, accelerates their death and prevents them from moving around too much. They even did an experiment with mice and found that CCAR1 is very important for the growth of tumors. The study concludes that Ccar1 acts as a partner of β-catenin and is important for maintaining the pluripotency of stem cells in the basal state. It emphasizes the importance of the Ccar1-β-catenin interaction in the regulation of pluripotency genes and suggests that targeting this interaction could have implications for stem cell research and regenerative medicine [11].

**Fig. 4:**
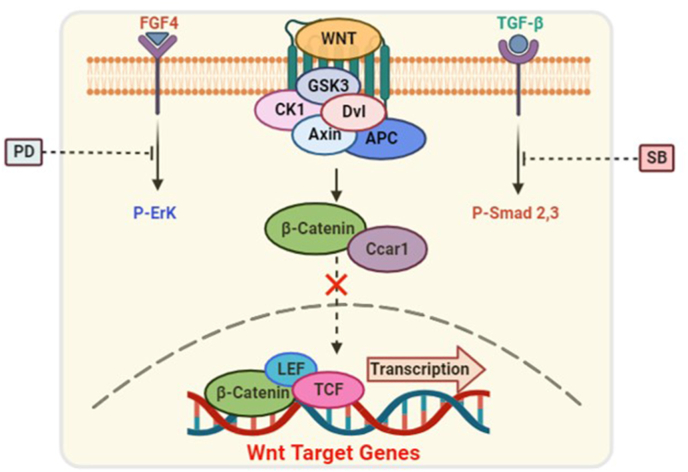
Ccar1 could be involved in the maintenance of mESCs' pluripotency through the interaction with β-catenin and prevents its nuclear translocation.

Taken together, our findings indicate that increasing *Ccar1* expression under R2i culture conditions can prevent β-catenin nuclear translocation and maintain the pluripotency and self-renewal of mESCs. This suggests that targeting Ccar1 could be a potential strategy to modulate stem cell fate and enhance their pluripotent properties (Fig. 4).

## 5 Limitations

Our study infers Ccar1–β-catenin interaction/function indirectly (siRNA, localization, and target-gene responses under R2i); we did not perform Co-IP/PLA or reporter assays, which will be a priority for future work.

## 6 Conclusion

The knockdown of *Ccar1* in R2i conditions resulted in the translocation of β-catenin into the nucleus and subsequent activation of Wnt target genes. This suggests that Ccar1 might regulate the localization and function of β-catenin in mouse embryonic stem cells. Additionally, the reduction in *Ccar1* expression led to decreased levels of pluripotency marker genes such as *Nanog* and *Rex1*. This implies that the interaction between Ccar1 and β-catenin could be involved in maintaining the self-renewal and pluripotency of mouse embryonic stem cells.

## Ethical statements

The experiments performed in this manuscript followed the no: IR.ACECR.ROYAN.REC.1394.78 approved by the Institutional Ethical Committee at Royan Institute.

## Declaration of competing interest

The authors declare that they have no known competing financial interests or personal relationships that could have appeared to influence the work reported in this paper.

## Data Availability

Data will be made available on request.
